# The mitochondrial genome of *Sapromyza sexpunctata* (Diptera: Lauxaniidae) and its phylogenetic analysis

**DOI:** 10.1080/23802359.2026.2732553

**Published:** 2026-09-15

**Authors:** Bei Zhou, Yuli Zhang, Hao Liu, Jia Han, Shengjuan Zhao, Wenliang Li

**Affiliations:** ^a^ Laboratory of Insect Evolution and Systematics, College of Horticulture and Plant Protection, Henan University of Science and Technology, Luoyang, China; ^b^ Henan Province Engineering Technology Research Center of Green Plant Protection, Luoyang, China; ^c^ Henan Key Laboratory of Insect Biology, Luoyang, China; ^d^ College of Food & Bioengineering, Henan University of Science and Technology, Luoyang, China

**Keywords:** Molecular systematics, Lauxaniinae, maximum likelihood

## Abstract

This study sequenced the complete mitochondrial genome of *Sapromyza sexpunctata* (Diptera: Lauxaniidae). Maximum-likelihood phylogenetic analysis supports Lauxanioidea monophyly and places this species sister to *Pachycerina*. The 16,671 bp genome comprises 37 genes (13 PCGs, 22 tRNAs, rrnL, and rrnS) and a control region, exhibiting a 77.54% A + T bias. Notably, *COX1* uses a putative atypical CGA start codon, and *ND3* has an incomplete T stop codon. These results provide a vital genetic reference for future Lauxaniidae systematic studies.

## Introduction

The genus *Sapromyza* Fallén (Diptera: Lauxaniidae) represents the second most species-rich genus within the family, exhibiting a broad distribution across major zoogeographical regions (Papp et al. 1998; Shatalkin 2000). Notably, despite being a crucial representative lineage of the subfamily Lauxaniinae, its mitogenomic sequences remain completely unreported among the 19 available for this family. *Sapromyza sexpunctata* Meigen 1826 is widely distributed throughout the Palearctic region. In this study, we sequenced the complete mitochondrial genome of *Sapromyza sexpunctata* for the first time. The newly generated genomic data enrich the molecular databases, laying a solid foundation for future research and holding significant implications for elucidating the phylogenetic position of the Lauxaniinae.

## Materials and methods

**Figure 1.**
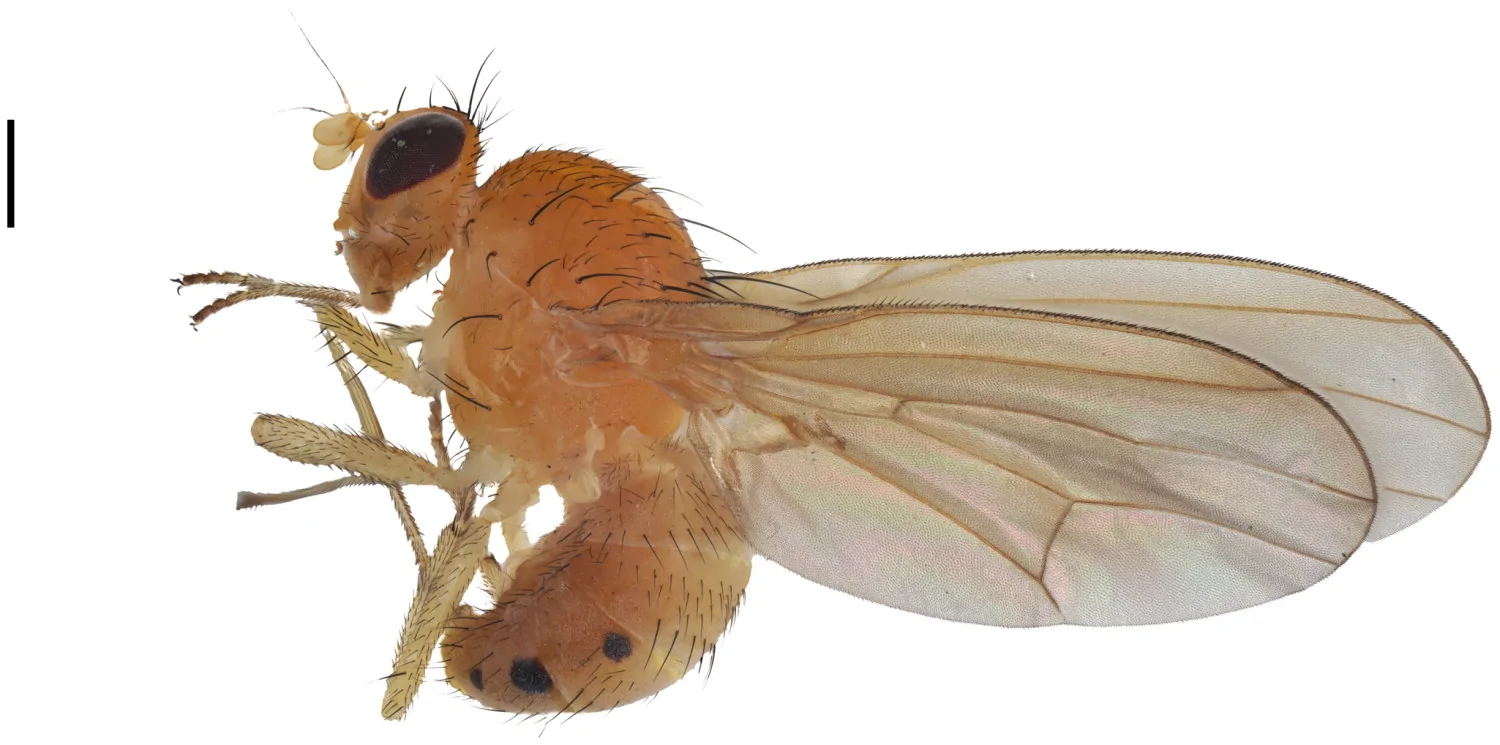
Reference image of the specimen, habitus, lateral view. (diagnosis. Body yellow, the mesonotum bearing 0 + 3 dorsocentral setae, and the anterior margins of abdominal tergites IV, V, and VI each featuring a pair of circular black spots). Scale bar = 0.5 mm.

Specimens of *Sapromyza sexpunctata* Meigen 1826 were collected from Haoyunsigou, Menyuan County, Qinghai Province, China (100.55° E, 37.13° N) on July 26, 2020. The specimens used in this study were not endangered or protected, and no specific ethical approval or collection permit was required. Morphological observations were conducted using a Motic SMZ-168 stereomicroscope. For genitalia preparation, the terminal portion of the abdomen was detached and macerated in a cold saturated NaOH solution for 6 h. It was subsequently rinsed and neutralized with water for dissection and examination. After observation in glycerin, the dissected genitalia were transferred to fresh glycerin and stored in a microvial pinned directly below the specimen. The specimen was identified by Wenliang Li. *Sapromyza sexpunctata* can be distinguished from congeneric species by the following characteristics: body yellow, the mesonotum bearing 0 + 3 dorsocentral setae (or bristles), and the anterior margins of abdominal tergites IV, V, and VI each featuring a pair of circular black spots (Shatalkin 2000). Adult images of *Sapromyza sexpunctata* were captured using a Canon EOS 6D camera (Canon, Tokyo, Japan) attached to a microscope, and the images were stacked using Helicon Focus v7.0.2.0 (Helicon Soft, Kharkiv, Ukraine) (Figure 1). To ensure reliable identification and exclude confusion with related species, the male genitalia were also photographed (Figure S1). The voucher specimen was preserved in absolute ethanol and deposited at Henan University of Science and Technology (www.haust.edu.cn), Henan Province, China (Contact person: Bei Zhou; Email: 240320191162@stu.haust.edu.cn) under the voucher number Lau202000005.

Total genomic DNA was extracted from the whole bodies (including heads and wings), and sequencing was performed by Tsingke Biotechnology Co., Ltd. (Tianjin, China). After DNA quality evaluation, a sequencing library with an insert size of 350 bp was constructed using the Nextera XT DNA library preparation kit (Illumina, San Diego, CA), followed by paired-end sequencing (150 bp) on the Illumina Novaseq 6000 platform. Raw data were filtered by the fastp (https://github.com/OpenGene/fastp) to remove reads with > 5% N base, reads with > 50% low-quality bases (*Q* ≤ 5), and adapter contamination. This process yielded 14.92 Gb of effective clean data, with Q20 and Q30 scores of 99.85% and 98.99%, respectively. Clean reads were *de novo* assembled using SPAdes v.3.14.1 with k-mer sizes 21, 45, 65, 85, and 105 (Bankevich et al. 2012). The resulting assembly graph was visualized with Bandage to verify the circularization of the mitogenome (Wick et al. 2015). To further verify the assembly accuracy, clean reads were mapped back to the assembled mitogenome using BWA, and the average sequencing depth was calculated as 3951.33× using SAMtools (Li 2013) (Figure S2). Despite uneven depth in the control region, its integrity was verified by the unambiguous assembly path in Bandage. The complete mitogenome was annotated using MITOS (http://mitos.bioinf.uni-leipzig.de/index.py). Ambiguous gene boundaries were resolved by manually correcting the start and stop codons. Finally, the circular map was generated using Organellar Genome DRAW v1.2 (Greiner et al. 2019).

Phylogenetic analysis was conducted using the 13 protein-coding genes (PCGs) and 2 ribosomal RNA genes of *Sapromyza sexpunctata* alongside 18 additional sequences downloaded from GenBank (Table S2). These included 10 Lauxanioidea species, two Tephritoidea species, two Opomyzoidea species, one Tabanoidea species, one Muscoidea species and two Ephydroidea species. Sequences were aligned *via* ClustalW in MEGA12; columns with gaps or missing data in > 50% of taxa were trimmed to remove hypervariable regions. An unpartitioned maximum-likelihood tree was reconstructed using IQ-TREE, based on the GTR+F + R3 model selected by ModelFinder *via* the Bayesian Information Criterion (BIC). Node support was evaluated using 1,000 ultrafast bootstrap replicates.

## Results

**Figure 2.**
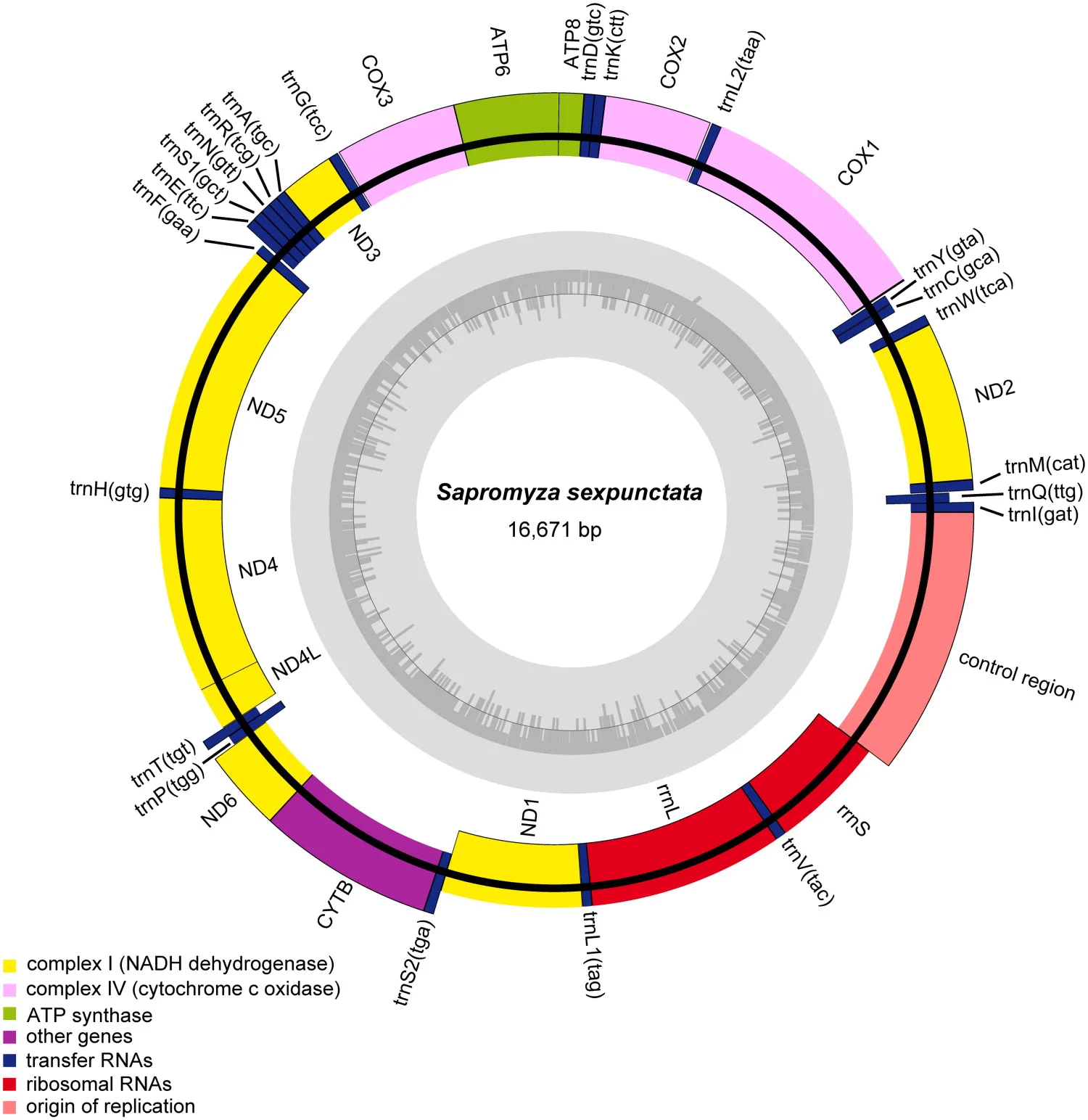
Circular map of the mitochondrial genome of *Sapromyza sexpunctata*. Genes located inside the circle are transcribed in a counter-clockwise direction, while those outside are transcribed clockwise. Different functional genes are color-coded. The inner grey histogram represents the GC content across the genome, with the Central grey line indicating the 50% threshold.

The complete mitochondrial genome of *Sapromyza sexpunctata* is approximately 16,671 bp in length (Figure 2). The nucleotide composition is 39.13% A, 12.97% C, 9.48% G, and 38.41% T, exhibiting a strong A + T bias. It contains the typical 37 genes commonly found in insect mitogenomes and features a complete control region. Despite belonging to different genera, the gene arrangement and functions in *Sapromyza sexpunctata* are highly conserved and similar to those of other known Lauxaniinae mitogenomes.

Among the 13 protein-coding genes (PCGs), four genes (*ND2*, *ND3*, *ND6*, and *ATP8*) utilize ATT as the start codon, while seven genes (*COX2*, *COX3*, *ND4*, *ND4L*, *ND5*, *ATP6*, and *CYTB*) initiate with ATG. Additionally, the *COX1* gene utilizes a putative atypical CGA initiation codon, and *ND1* begins with TTG. Regarding termination codons, 11 PCGs (*ATP6*, *ATP8*, *COX1*, *COX2*, *COX3*, *ND1*, *ND2*, *ND4L*, *ND5*, *ND6*, and *CYTB*) terminate with the standard TAA stop codon. *ND3* ends with an incomplete

stop codon T + tRNA, and one gene *ND4* utilizes a TAG stop codon. The rrnS and rrnL genes are 787 bp and 1,329 bp in length, respectively. Gene overlaps range from 1 to 7 bp (longest between *ATP6*/*ATP8* and *ND4*/*ND4L*) (Table S1).

**Figure 3.**
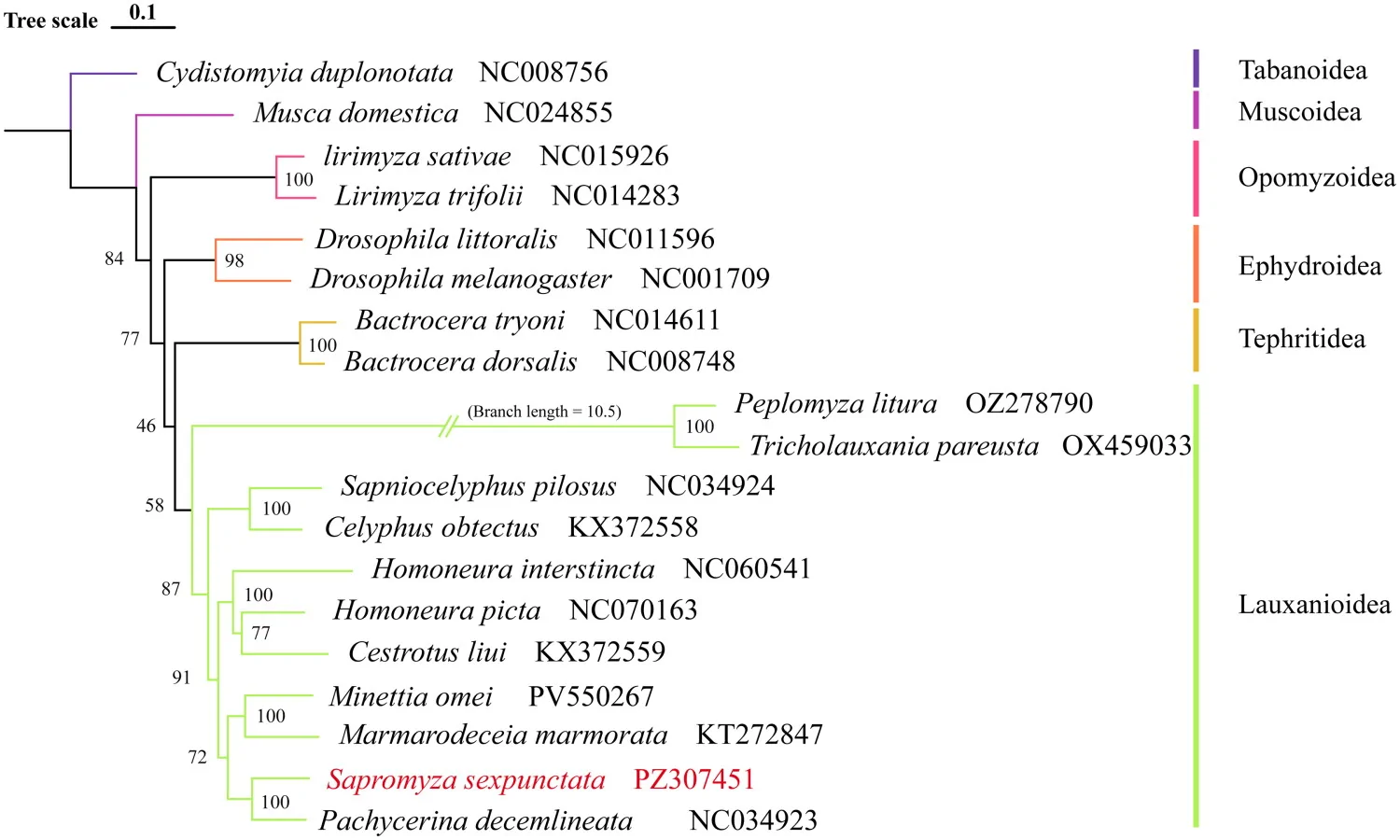
Maximum-likelihood phylogenetic tree based on 13 PCGs (third codon positions excluded) and 2 ribosomal genes. Node numbers indicate ultrafast bootstrap values; colored branches and vertical bars represent respective superfamilies. The following sequences were used: PZ307451 (this study), PV550267 (Zhang et al. 2025), KX372559, NC034923, NC034924, KX372558 (Li et al. 2017), NC070163 (Yao et al. 2024), NC008756 (Shi et al. 2007), NC024855 (Li et al. 2016), NC015926 (Fei et al. 2011), NC014283 (Wang et al. 2011), NC001709 (Lewis et al. 1995), OX459033 (Falk 2024), NC008748 (Yu et al. 2007), NC014611 (Nardi et al. 2010), KT272847 (Junqueira et al. 2016), NC011596 (Andrianov et al. 2010), NC060541 (unpublished), OZ278790 (unpublished).

Maximum Likelihood phylogenetic analysis supports the monophyly of the superfamily Lauxanioidea. The overarching topology of the phylogenetic tree is recovered as follows: Tabanoidea + (Muscoidea + (Opomyzoidea + (Ephydroidea + (Tephritoidea + Lauxanioidea)))). Within the superfamily Lauxanioidea, the internal topology is resolved as: (*Sapromyza* + *Pachycerina*) + (*Minettia* + *Marmarodeceia*) + (*Homoneura* + *Cestrotus*) + (*Spaniocelyphus* + *Celyphus*) + (*Peplomyza* + *Tricholauxania*). Furthermore, the family Celyphidae is nested within Lauxaniidae, and the monophyly of Lauxaniidae has not been supported by phylogenetic studies (Figure 3).

## Discussion and conclusion

We report the first complete mitochondrial genome of *Sapromyza sexpunctata*. It exhibits typical dipteran features (significant A + T bias, conserved gene arrangement, incomplete stop codons) and utilizes a putative atypical CGA start codon for *COX1*. Maximum-likelihood phylogenetic analysis supports traditional morphology, placing *Sapromyza sexpunctata* within the monophyletic Lauxanioidea. The resulting internal topology, (*Sapromyza* + *Pachycerina*) + (*Minettia* + *Marmarodeceia*) + (*Homoneura* + *Cestrotus*) + (*Spaniocelyphus* + *Celyphus*) + (*Peplomyza* + *Tricholauxania*), offers new molecular validation for existing morphological hypotheses.

Despite being the most diverse Lauxaniinae genus, the phylogeny of *Sapromyza* remains controversial, largely due to the lack of phylogenetic analyses with adequate species coverage, particularly with respect to species from different geographic regions. Consequently, the mitogenome of *Sapromyza sexpunctata* reported herein contributes to our understanding of the evolutionary relationships of this species, establishes an important genetic database for subsequent molecular evolutionary studies, and represents an important step toward resolving the phylogenetic relationships of Lauxaniidae. Future studies will obtain samples of more species from different geographic regions and high-quality mitogenomic data to systematically resolve the phylogenetic positions of *Sapromyza* species using mitochondrial DNA markers.

## Supplementary Material

Supplementary_Material.docx

## Data Availability

The genome sequence data that support the findings of this study are openly available in GenBank of NCBI at https://www.ncbi.nlm.nih.gov, reference number PZ307451. The associated BioProject, SRA, and BioSample numbers are PRJNA1480359, SRR39244030, and SAMN61026148, respectively.

## References

[CIT0001] Andrianov B et al. 2010. Comparative analysis of the mitochondrial genomes in *Drosophila virilis* species group (Diptera: Drosophilidae). Trends Evol Biol. 2(1):e4. 10.4081/eb.2010.e4

[CIT0002] Bankevich A et al. 2012. SPAdes: a new genome assembly algorithm and its applications to single-cell sequencing. J Comput Biol. 19(5):455–477. 10.1089/cmb.2012.0021 22506599 PMC3342519

[CIT0003] Falk S. 2024. Darwin tree of life consortium. The genome sequence of a lauxaniid fly. Wellcome Open Res. 9:586. 10.12688/wellcomeopenres.23099.1 39563950 PMC11574340

[CIT0004] Greiner S, Lehwark P, Bock R. 2019. OrganellarGenomeDRAW (OGDRAW) version 1.3.1: expanded toolkit for the graphical visualization of organellar genomes. Nucleic Acids Res. 47(W1):W59–W64. 10.1093/nar/gkz238 30949694 PMC6602502

[CIT0005] Junqueira ACM et al. 2016. Large-scale mitogenomics enables insights into Schizophora (Diptera) radiation and population diversity. Sci Rep. 6(1):21762–21774. 10.1038/srep21762 26912394 PMC4766414

[CIT0006] Lewis DL, Farr CL, Kaguni LS. 1995. *Drosophila melanogaster* mitochondrial DNA: completion of the nucleotide sequence and evolutionary comparisons. Insect Mol Biol. 4(4):263–278. 10.1111/j.1365-2583.1995.tb00032.x 8825764

[CIT0007] Li H. 2013. Aligning sequence reads, clone sequences and assembly contigs with BWA-MEM. Arxiv Preprint Arxiv. 1303.3997

[CIT0008] Li X et al. 2017. Mitochondrial genomes provide insights into the phylogeny of Lauxanioidea (Diptera: cyclorrhapha). Int J Mol Sci. 18(4):773–795. 10.3390/ijms18040773 28420076 PMC5412357

[CIT0009] Li XK et al. 2016. The complete mitochondrial genomes of *Musca domestica* and *Scathophaga stercoraria* (Diptera: Muscoidea: Muscidae and Scathophagidae). Mitochondrial DNA Part A: DNA Mapping, Sequencing, and Analysis. 27(2):1435–1436. 10.3109/19401736.2014.953095 25163032

[CIT0010] Meigen JW. 1826. Systematische Beschreibung der bekannten europäischen zweiflügeligen Insekten. Fünfter Theil. Schulz-Wundermann.

[CIT0011] Nardi F et al. 2010. Domestication of olive fly through a multi-regional host shift to cultivated olives: comparative dating using complete mitochondrial genomes. Mol Phylogenet Evol. 57(2):678–686. 10.1016/j.ympev.2010.08.008 20723608

[CIT0012] Papp L, Shatalkin AI, Papp L. 1998. Family Lauxaniidae. In: Darvas B, editors. Contributions to a manual of palaearctic diptera; Vol. 3. Budapest: Science Herald Publishers, pp. 383–400.

[CIT0013] Semelbauer M. 2016. Molecular phylogeny of lauxaniid flies (Diptera, Cyclorrhapha) confirms non-monophyly of *Sapromyza* Fallen 1810. Insect Syst Evol. 47(4):389–409. 10.1163/1876312X-47032148

[CIT0014] Shatalkin AI. 2000. Keys to the Palaearctic flies of the family Lauxaniidae (Diptera). Zoologicheskie Issledovania. 5:1–102.

[CIT0015] Shi L, Li WL, Yang D. 2012. Note on seventeen species from China of the genus *Sapromyza*, with description of two new species (Diptera, Lauxaniidae). Acta Zootaxonomica Sinica. 37:185–198. 10.3724/SP.J.1141.2012.02185

[CIT0016] Wang SY et al. 2011. The complete mitochondrial genome of the leafminer *Liriomyza trifolii* (Diptera: Agromyzidae). Mol Biol Rep. 38(2):687–692. 10.1007/s11033-010-0156-5 20376704

[CIT0017] Wick RR, Schultz MB, Zobel J, Holt KE. 2015. Bandage: interactive visualization of de novo genome assemblies. Bioinformatics. 31(20):3350–3352. 10.1093/bioinformatics/btv383 26099265 PMC4595904

[CIT0018] Yang F, Du YZ, Wang LP, Cao JM, Yu WW. 2011. The complete mitochondrial genome of the leafminer *Liriomyza sativae* (Diptera: Agromyzidae): Great difference in the A + T-rich region compared to *Liriomyza trifolii*. Gene. 485(1):7–15. 10.1016/j.gene.2011.05.031 21703334

[CIT0019] Yao Y et al. 2024. The mitochondrial genome of *Homoneura picta* (Diptera: Lauxaniidae) and its phylogenetic analysis. Mitochondrial DNA B Resour. 9(6):828–831. 10.1080/23802359.2024.2333560 38919812 PMC11198121

[CIT0020] Yu DJ, Xu L, Nardi F, Li JG, Zhang RJ. 2007. The complete nucleotide sequence of the mitochondrial genome of the oriental fruit fly, *Bactrocera dorsalis* (Diptera: Tephritidae). Gene. 396(1):66–74. 10.1016/j.gene.2007.02.023 17433576

[CIT0021] Zhang Y et al. 2025. The mitochondrial genome of *Minettia omei* (Diptera: Lauxaniidae) and its phylogenetic analysis. Mitochondrial DNA B Resour. 10(11):1016–1020. 10.1080/23802359.2025.2569539 41069866 PMC12507106

