# Supplementary material for: The mitochondrial genome of Sapromyza sexpunctata (Diptera: Lauxaniidae) and its phylogenetic analysis
Source: Mitochondrial DNA B Resour. 2026 Sep 15;11(10):1157–61. doi: 10.1080/23802359.2026.2732553 (PMC13580367; doi:10.1080/23802359.2026.2732553)
Supplement: Supplementary file 1 [file TMDN_A_2732553_SM6528.docx]

**Table S1.** Organization of the mitogenome of *Sapromyza sexpunctata*

| Start | End | Length | Strand | Gene |
| --- | --- | --- | --- | --- |
| 1 | 66 | 66 | + | trnI(gat) |
| 64 | 132 | 69 | - | trnQ(ttg) |
| 139 | 207 | 69 | + | trnM(cat) |
| 208 | 1231 | 1024 | + | ND2 |
| 1232 | 1299 | 68 | + | trnW(tca) |
| 1409 | 1472 | 64 | - | trnC(gca) |
| 1474 | 1539 | 66 | - | trnY(gta) |
| 1550 | 3080 | 1531 | + | COX1 |
| 3081 | 3145 | 65 | + | trnL2(taa) |
| 3155 | 3842 | 688 | + | COX2 |
| 3843 | 3913 | 71 | + | trnK(ctt) |
| 3913 | 3978 | 66 | + | trnD(gtc) |
| 3979 | 4140 | 162 | + | ATP8 |
| 4134 | 4811 | 678 | + | ATP6 |
| 4811 | 5599 | 789 | + | COX3 |
| 5608 | 5672 | 65 | + | trnG(tcc) |
| 5673 | 6024 | 352 | + | ND3 |
| 6025 | 6089 | 65 | + | trnA(tgc) |
| 6089 | 6151 | 63 | + | trnR(tcg) |
| 6152 | 6217 | 66 | + | trnN(gtt) |
| 6218 | 6284 | 67 | + | trnS1(gct) |
| 6285 | 6349 | 65 | + | trnE(ttc) |
| 6368 | 6434 | 67 | - | trnF(gaa) |
| 6435 | 8171 | 1737 | - | ND5 |
| 8172 | 8237 | 66 | - | trnH(gtg) |
| 8238 | 9576 | 1339 | - | ND4 |
| 9570 | 9866 | 297 | - | ND4L |
| 9869 | 9934 | 66 | + | trnT(tgt) |
| 9935 | 10000 | 66 | - | trnP(tgg) |
| 10003 | 10527 | 525 | + | ND6 |
| 10527 | 11662 | 1136 | + | CYTB |
| 11663 | 11729 | 67 | + | trnS2(tga) |
| 11730 | 12693 | 964 | - | ND1 |
| 12695 | 12758 | 64 | - | trnL1(tag) |
| 12759 | 14087 | 1329 | - | rrnL |
| 14088 | 14159 | 72 | - | trnV(tac) |
| 14160 | 14946 | 787 | - | rrnS |
| 14947 | 16671 | 1725 | + | D-loop |

**Table S2.** Taxa, taxonomic placements, GenBank accession numbers, and references for the sequences used in the phylogenetic analysis.

| Species | Taxonomic placement | GenBank accession | Reference |
| --- | --- | --- | --- |
| *Cydistomyia duplonotata* | Tabanoidea | NC008756 | Shi et al. 2007 |
| *Musca domestica* | Muscoidea | NC024855 | Li et al. 2016 |
| *Lirimyza trifolii* | Opomyzoidea | NC014283 | Wang et al. 2011 |
| *lirimyza sativae* | Opomyzoidea | NC015926 | Fei et al. 2011 |
| *Drosophila melanogaster* | Ephydroidea | NC001709 | Lewis et al. 1995 |
| *Drosophila littoralis* | Ephydroidea | NC011596 | Andrianov et al. 2010 |
| *Bactrocera dorsalis* | Tephritidea | NC008748 | Yu et al. 2007 |
| *Bactrocera tryoni* | Tephritidea | NC014611 | Nardi et al. 2010 |
| *Tricholauxania pareusta* | Lauxanioidea | OX459033 | Falk et al. 2024 |
| *Peplomyza litura* | Lauxanioidea | OZ278790 | GenBank (Unpublished) |
| *Celyphus obtectus* | Lauxanioidea | KX372558 | Li et al. 2017 |
| *Sapniocelyphus pilosus* | Lauxanioidea | NC034924 | Li et al. 2017 |
| *Homoneura interstincta* | Lauxanioidea | NC060541 | GenBank (Unpublished) |
| *Cestrotus liui* | Lauxanioidea | KX372559 | Li et al. 2017 |
| *Homoneura picta* | Lauxanioidea | NC070163 | Yao et al. 2024 |
| *Marmarodeceia marmorata* | Lauxanioidea | KT272847 | Junqueira et al. 2016 |
| *Minettia omei* | Lauxanioidea | PV550267 | Zhang et al. 2024 |
| *Pachycerina decemlineata* | Lauxanioidea | NC034923 | Li et al. 2017 |
| *Sapromyza sexpunctata* | Lauxanioidea | PZ307451 | This study |

**Figure S1**. Male genitalia of *Sapromyza sexpunctata* Meigen, 1826. A. Aedeagal complex, ventral view; B. Aedeagal complex, lateral view. Scale bar = 0.1 mm.

**Figure S2.** Coverage depth plot of the *Sapromyza sexpunctata* mitochondrial genome. The x-axis represents the length of the mitochondrial genome, and the y-axis represents the coverage depth.
